# Characterization of Low Molecular Weight Sulfate Ulva Polysaccharide and its Protective Effect against IBD in Mice

**DOI:** 10.3390/md18100499

**Published:** 2020-09-29

**Authors:** Yuanyuan Li, Han Ye, Ting Wang, Peng Wang, Ruizhi Liu, Yinping Li, Yingying Tian, Jingliang Zhang

**Affiliations:** 1 College of Food Science and Engineering, Ocean University of China, Qingdao 266003, China; yuanyuan032220@163.com (Y.L.); 17852720293@163.com (H.Y.); Wang_xx0419@163.com (T.W.); pengwang@ouc.edu.cn (P.W.); 2 State Environmental Protection Key Laboratory of Estuarine and Coastal Environment, Chinese Research Academy of Environmental Sciences, Beijing 100012, China; 3 Shandong Provincial Key Laboratory of Biochemical Engineering, College of Marine Science and Biological Engineering, Qingdao University of Science and Technology, Qingdao 266042, China; 4 Marine Biomedical Research Institute of Qingdao, Qingdao 266071, China; 15704317507@163.com (Y.T.); yupinghui7373262@126.com (J.Z.)

**Keywords:** *Ulva pertusa*, polysaccharides, colitis, anti-inflammatory, antioxidant

## Abstract

Inflammatory bowel disease (IBD) has been gradually considered a public health challenge worldwide. Sulfated polysaccharides, extracted from seaweed, have been shown to have an anti-inflammatory effect on the disease. In this study, LMW-ulvan, a unique sulfate Ulva polysaccharide with low molecular weight, was prepared using the enzymatic method. The structural characterization of LMW-ulvan and its protective effect on colitis induced by dextran sulfate sodium (DSS) were studied. The results showed that LMW-ulvan with molecular weight of 2.56 kDa consists of 57.23% rhamnose (Rha), 28.76% xylose (Xyl), 7.42% glucuronic acid (GlcA), and 1.77% glucose (Glc). Its backbone contains (1→3,4)-linked Rha, (1→4)-linked Xyl, and (1→4)-linked GlcA with small amounts of (1→4)-linked Rha residues; sulfate substitution was at C-3 of Rha. LMW-ulvan was found to reduce DSS-induced disease activity index, colon shortening, and colonic tissue damage, which were associated with decreased oxidative stresses and inflammation, thus improving the expression of tight junction proteins. These results indicate that LMW-ulvan is able to improve colitis and may be a promising application for IBD.

## 1. Introduction

Ulcerative colitis (UC), a significant form of inflammatory bowel disease (IBD), is a chronic inflammatory disorder of the colonic mucosa. It usually starts in the rectum and spreads to the colon proximally, in a continuous manner [1]. The global prevalence of IBD is increasing every year—more than 0.3% of the population suffers from the disease [2]. The pathogenesis of IBD is unclear; however, many researches claim that it is a result of genetic defects, unhealthy lifestyles, imbalance of intestinal microbiota, or immune dysbiosis [3,4,5]. Typical features of UC include structural and functional impairment of the intestinal mucosa, accompanied by diarrhea, rectal bleeding, and other symptoms [6]. Currently, surgery and drugs (including corticosteroids, aminosalicylic acid, and immunosuppressive agents) are the main strategies from a clinical aspect [7]. However, in addition to the high costs and incomplete treatment issues, these drug interventions have certain side-effects and have been seen to lose efficacy in long-term use patients [8]. Consequently, more efforts should be made to enrich treatment and disease prevention methods.

Numerous reports have testified to inflammatory reactions being a crucial factor causing UC [9]. The cytokines secreted by intestinal epithelial cells are important markers of the intestinal immune system and regulate their inflammatory responses [10]. Pro-inflammatory cytokines such as tumor necrosis factor (TNF)-α, interferon (IFN)-γ, and interleukin (IL)-1β can promote an inflammatory response, eventually causing colon tissue injury, while anti-inflammatory cytokines such as IL-4 and IL-10 can reduce inflammation [11]. Excessive pro-inflammatory cytokines increase the production of reactive oxygen species (ROS), which ultimately cause intestinal epithelial cell injury and aggravate pathogenesis [12]. Hence, materials with the ability to recover abnormal levels of cytokines and oxidative stress may be promising therapeutics for IBD.

Ulvan is a kind of sulphated polysaccharide located in the cell walls of *Ulva*; it represents an abundant marine resource distributed worldwide. Ulvan extracted from different species of *Ulva* consists primarily of Rha (16.8–45.0%), GlcA (6.5–19.0%), Xyl (2.1–12.0%), iduronic acid (IdoA) (0.7–9.1%), Glc (0.5–6.8%), and sulphate (14.3–23.2%) and its backbone is mostly composed of α-1,4- and α-1,2,4- linked L-rhamnose, β-1,4- and terminally linked D-glucuronic acid and β-1,4-linked D-xylose [13]. Like other sulfated polysaccharides, ulvan exerts various therapeutic activities, such as antibacterial, immunostimulatory, antitumor, antioxidant, antihyperlipidemic, and anticoagulant properties [14,15]. Qi et al. (2005) reported that high sulfate content ulvan showed a stronger scavenging activity of superoxide and hydroxyl radicals, reducing power, and metal chelating ability [16]. In a research work by Li et al. (2018), the ulvan extracted from *Ulva pertusa* showed significant protection against liver damage by oxidative stress induced by a cholesterol-rich diet [17]. De Araújo et al. (2016) found that the enzymatic digestion of ulvan extracted from *Ulva lactuca* had a vascular anti-inflammatory effect by decreasing TNF-α and IL-1 levels [18]. Additionally, Berri et al. (2017) reported that ulvan extracted from *Ulva armoricana* was able to initiate and amplify the protective immune responses of the host, and regulate mucosal immunity against intestinal pathogens [19].

Reports on the antiinflammatory and antioxidant effects of ulvan are readily available, but few reports have been published on its protective effects in rats with DSS-induced colitis. In this study, LMW-ulvan produced by the enzymatic method was purified and characterized, and its potential to alleviate IBD symptoms in DSS-induced mice and protect the intestinal epithelial barrier were investigated.

## 2. Results and Discussion

### 2.1. Characterization of LMW-Ulvan

#### 2.1.1. Determination of Molecular Weight of LMW-Ulvan

**Figure 1:**
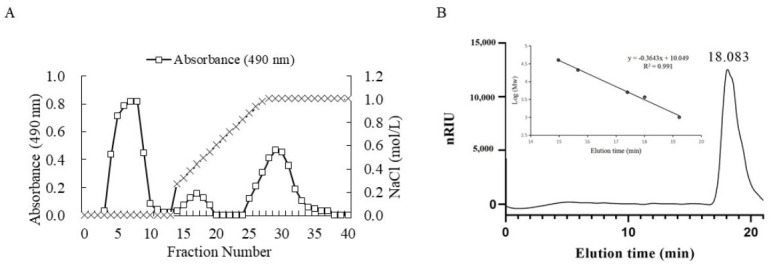
Purification of LMW-ulvan using a DEAE column (**A**) and its gel permeation chromatogram (**B**).

The average molecular weight of ulvan was 1068.2 kDa according to a previous research by Chi et al. (2020) [20]. Because of its high molecular weight, ulvan usually shows poor biological absorption compared to oligosaccharide [13]. Consequently, the ulvan was degraded by ulvan lyase and the purified LMW-ulvan was prepared using ion exchange chromatography (Figure 1A). The molecular weight distribution of LMW-ulvan was then measured (Figure 1B). According to the standard curve obtained from dextran standards with different molecular weights (logMw = −0.3643x + 10.049, R^2^ = 0.991) and retention time of the polysaccharide peak, the weight-average molecular weight of LMW-ulvan was calculated to be 2.56 kDa.

#### 2.1.2. Monosaccharide Composition Analysis of LMW-Ulvan

**Figure 2:**
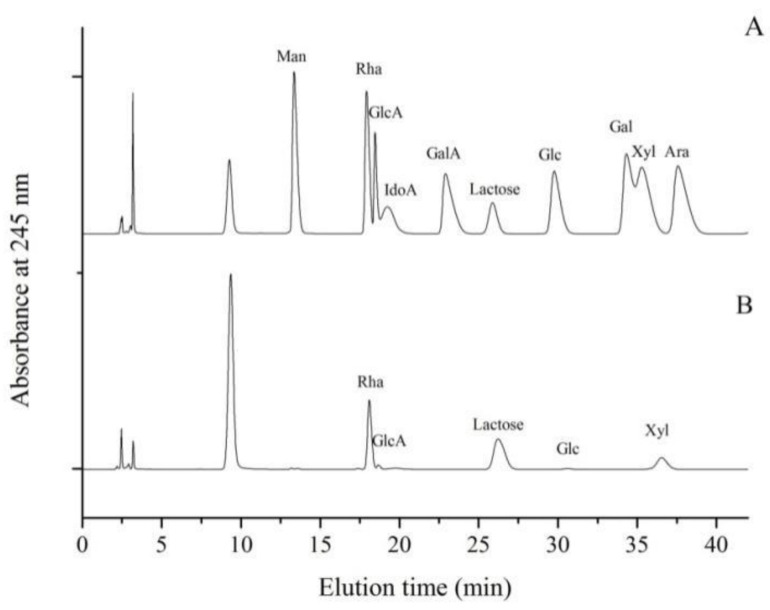
High performance liquid chromatography (HPLC) analyses of monosaccharide standards (**A**) and LMW-ulvan (**B**). Lactose is the internal standard.

The LMW-ulvan generated in this study was mainly composed of 57.23% Rha, 28.76% Xyl, 7.42% GlcA, and 1.77% Glc (Figure 2). The monosaccharide composition of LMW-ulvan was slightly different from that of ulvan, whose molar percentage of Rha, Xyl, GlcA, IdoA, and Glc was 56.50%, 20.34%, 16.95%, 2.82%, and 3.39%, respectively [20]. The lyase used in this study belonged to the PL25 family, which cleaves the (1→4) glycosidic bond between 3-*O*-sulfated Rha and GlcA [21]. After enzymatic hydrolysis, unsaturated uronic acid was formed in a β-elimination reaction [22]. Consequently, the characterization of ulvan lyase might be the reason for a significant decrease in percentage of GlcA.

#### 2.1.3. Methylation and Gas Chromatography-Mass Spectrometer (GC-MS) Analysis of LMW-Ulvan

The integrated structure of LMW-ulvan, including linkages and sulfate substitution position, were further determined by methylation analysis and GC-MS. The completeness of methylation of LMW-ulvan and desulfated LMW-ulvan (dsLMW-ulvan) was confirmed by FT-IR spectroscopy with the disappearance of OH bands (data not shown). As shown in Table 1, LMW-ulvan mainly consisted of (1→3,4)-linked Rha, and (1→4)-linked Xyl with small amounts of (1→4)-linked Rha residues. Sulfate substitution was at C-3 of (1→4)-linked Rha because of the distinctive increase of amounts of (1→4)-linked Rha and significantly decrease of the amounts of (1→3,4)-linked Rha in dsLMW-ulvan. Besides, according to the characterization of ulvan lyase belonged to the PL25 family, which cleaves the (1→4) glycosidic bond between 3-*O*-sulfated Rha and GlcA [21]. Thus we deduced that there is 4)-GlcAp-(1 in the LMW-ulvan. The linkages and sulfate substitution position of LMW-ulvan are similar to polysaccharide from *Ulva* species [23].

### 2.2. LMW-Ulvan Relieves the Manifestations of IBD Induced by DSS

**Figure 3:**
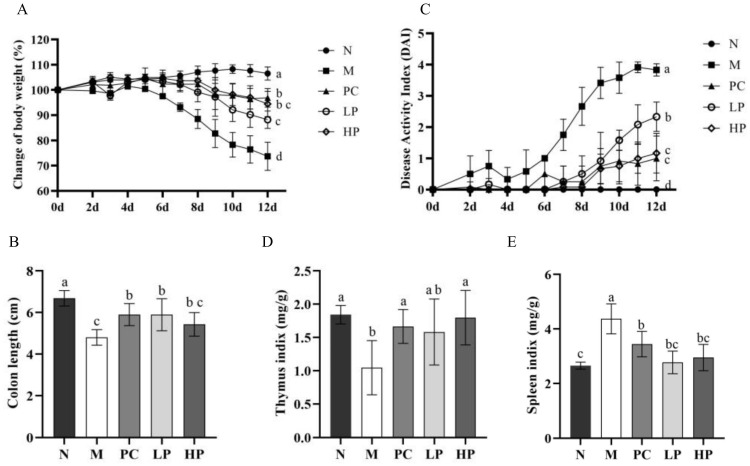
Assess the severity of IBD (Inflammatory bowel disease) in each group: (**A**) Change in body weight, (**B**) colon length of mice in each group, (**C**) DAI (disease activity index) score, (**D**) thymus index, and (**E**) spleen index. N: normal group, M: model, PC: positive control, LP: low dose LMW-ulvan (50 mg/kg), and HP: high dose LMW-ulvan (100 mg/kg).

To evaluate the anti-colitis effects of LMW-ulvan, the DSS was used to establish a model to mimic human UC and determine the therapeutic effect of LMW-ulvan [24]. Preliminary tests revealed that 30 mg/kg of LMW-ulvan showed no protective effects in IBD rats, while 50 mg/kg of LMW-ulvan began to show protective effects in IBD. Besides, 100 and 120 mg/kg of LMW-ulvan were more effective. Thus, 50 and 100 mg/kg were chosen for subsequent studies. The results of the acute toxicity experiment showed that an LMW-ulvan dose less than 1200 mg/kg showed no significant damage to ICR mice. Consequently, both doses used in this study were safe. The clinical symptoms of IBD in mice were comprehensively determined by changes in body weight (Figure 3A), colon length (Figure 3B), and DAI (disease activity index) score (Figure 3C). Body weight of the mice in the N group showed a gradual upward trend during the experiment period. In contrast, the body weight of the mice in the M group revealed a downward trend, especially after the fifth day. The loss in weight of the mice was significantly improved on treatment with LMW-ulvan of both dosages. There was no significant difference between the LP group and the PC group. Another indicator reflecting the severity of intestinal inflammation is colon length, which is restored with improvement in inflammation [25,26]. The colon length of mice in the M group was significantly shorter than that of the N group (Figure 3B). However, after treatment with LMW-ulvan, the colon was distinctly longer than that of the M group and similar to that of the PC group. In addition, the DAI score were also significantly reduced in the LMW-ulvan treatment group compared with that of the M group on the sixth day (Figure 3C). Spleen and thymus index was measured as an indirect marker of inflammation in all the experiment groups. As shown in Figure 3D,E, DSS decreased the thymus index and increased the spleen index significantly, reflecting on worse immune activity. LMW-ulvan was observed to improve the immune system because the spleen and thymus index of the HP group recovered to normal levels and showed no significant difference as compared to that of the N group. Besides, Figure 3E also showed that the LMW-ulvan of both dosages could significantly relieve splenomegaly induced by DSS.

### 2.3. LMW-Ulvan Reverses the Histological Injury of Colonic Epithelium Caused by DSS

**Figure 4:**
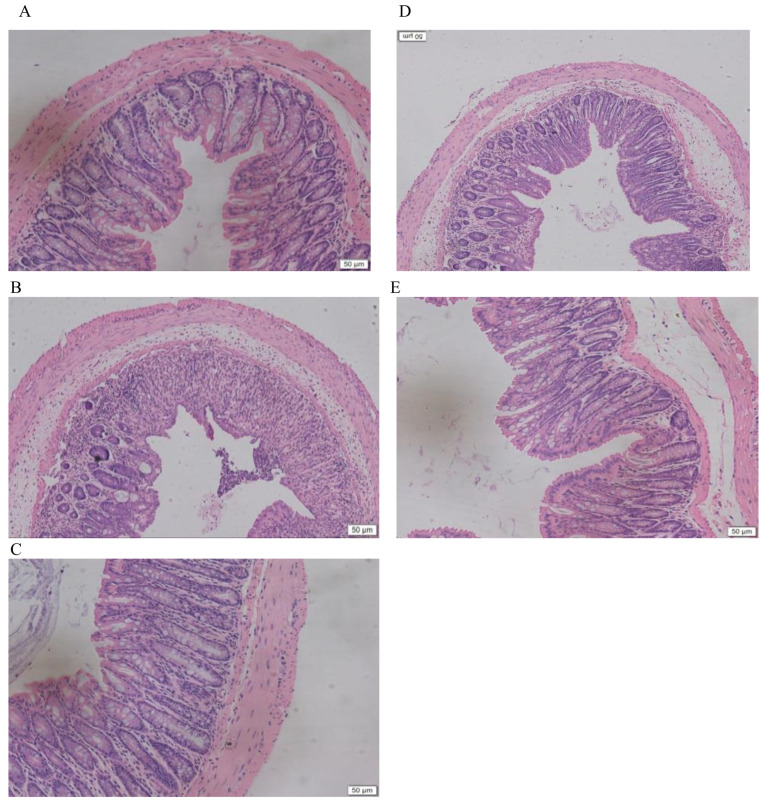
H&E staining of representative colons from different treatment groups (200×): (**A**) N group, (**B**) M group, (**C**) PC group, (**D**) LP group, and (**E**) HP group. N: normal group, M: model, PC: positive control, LP: low dose LMW-ulvan (50 mg/kg), and HP: high dose LMW-ulvan (100 mg/kg).

Due to severe colitis, mice in the M group exhibited mucosal degeneration and necrosis, distortion of crypts, loss of goblet cells, and mucosal and submucosal infiltration of mononuclear inflammatory cells, as compared to that of the N group (Figure 4A,B). However, in all mice in the LP group, infiltration and aggregation of mononuclear inflammatory cells were observed in the submucosa of the colon, with improvement observed in epithelial degeneration and necrosis. In mice from the HP group, a nearly intact structure with few inflammatory cells as that of the N group was exhibited. This result indicates that LMW-ulvan could reduce the inflammatory infiltration and damage caused by DSS.

### 2.4. Effects of LMW-Ulvan on Cytokines in Colon Tissue and Serum

**Figure 5:**
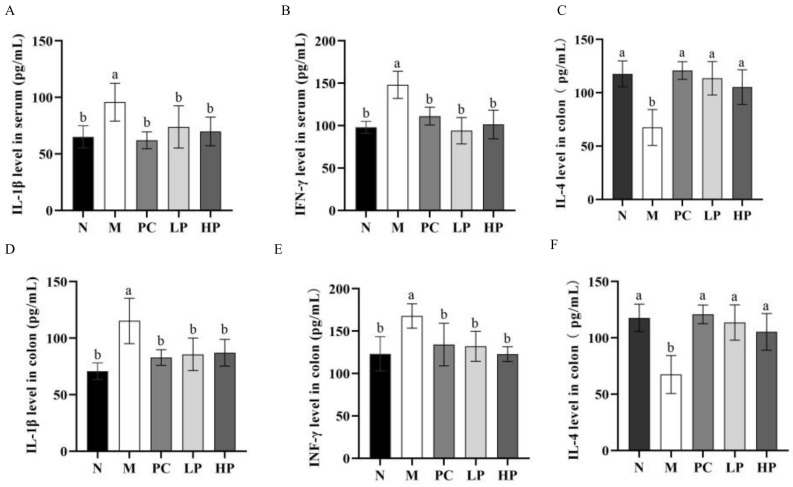
Levels of inflammatory cytokines in serum and colon tissues. (**A**) interleukin (IL)-1β level in serum. (**B**) IL-4 level in serum. (**C**) interferon (IFN)-γ level in serum. (**D**) IL-1β level in colon. (**E**) IL-4 level in colon. (**F**) IFN-γ level in colon. N: normal group, M: model, PC: positive control, LP: low dose LMW-ulvan (50 mg/kg), and HP: high dose LMW-ulvan (100 mg/kg).

It is well known that an abnormal inflammatory response is one of the most striking features of IBD [27]. The result showed that DSS increased the levels of IL-1β and IFN-γ significantly and decreased the level of IL-4, when compared to the N group (Figure 5). After administration of LMW-ulvan, the levels of IL-1β, IFN-γ, and IL-4 in colon tissue and serum all recovered to normal levels. IL-1β is a kind of pro-inflammatory cytokine and its active form is regulated by caspase 1 secreted from the NLRP3 inflammasome [28]. In this study, LMW-ulvan reduced the level of IL-1β in the colon and serum, which indicated its possibility to suppress NLRP3 inflammasome activation. Activated Th cells are classified as type 1 of T helper cells (Th1) and type 2 of T helper cells (Th2) according to different sorts and bioactivities of cytokines [29]. Usually, Th1 mediates a cell-mediated immune response, while Th2 mediates a humoral immune response [30]. IFN-γ and IL-4 are representative cytokines to coordinate immunity secreted by Th1 and Th2, respectively [31]. The IFN-γ/IL-4 ratio can also reflect the balance of Th1/Th2. The results showed that IFN-γ/IL-4 ratios in serum of the N, M, PC, LP, and HP groups were similar to that in the colon and were about 1.0, 2.5, 1.1, 1.2, and 1.1, respectively. Consequently, DSS disturbs the balance of Th1/Th2 toward to Th1 by increasing the value of IFN-γ and reducing the secretion of IL-4. The results indicated that LMW-ulvan could inhibit the Th1 cell response and improve the Th2 cell response, which is beneficial in alleviating the inflammatory damage caused by DSS.

### 2.5. Effects of LMW-Ulvan on Malondialdehyde (MDA), GPx, and Catalase (CAT) in Colonic Tissue

**Figure 6:**
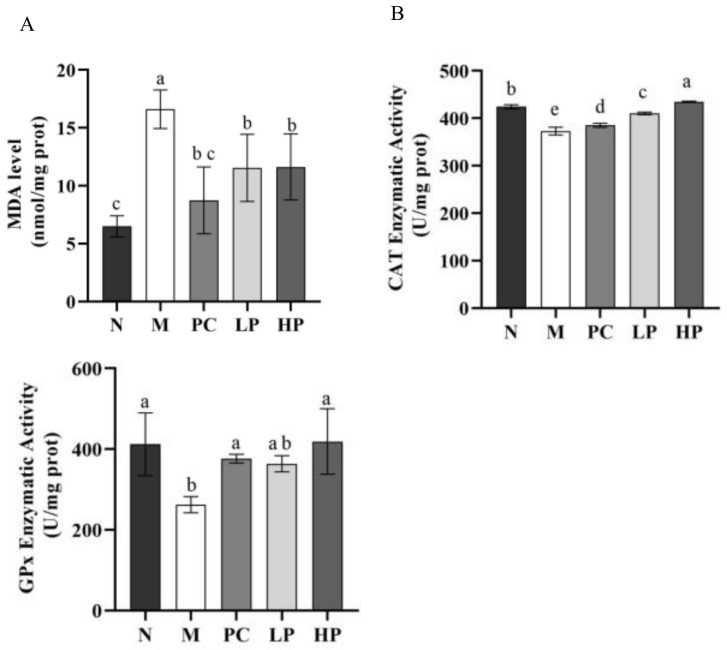
The level of MDA (**A**), activity of GPx (**B**), and activity of CAT(**C**). N: normal group, M: model, PC: positive control, LP: low dose LMW-ulvan (50 mg/kg), and HP: high dose LMW-ulvan (100 mg/kg).

Excessive inflammation augments oxidative stress, which has been associated with extensive infiltration of immune cells, and activation of NF-κB signaling and ER stress, thereby causing tissue damage and exacerbation of a patient’s condition [12,32,33]. As shown in Figure 6, DSS significantly enhanced the MDA level, but downregulated the enzyme activity of GPx and CAT. LMW-ulvan at doses of 50 and 100 mg/kg significantly reduced (*p* < 0.05) the MDA level and increased the enzyme activity of GPx and CAT. Specifically, the activity of GPx and CAT of the HP group was restored to that of the normal group. MDA, an indicator reflecting the accumulation of ROS in response to oxidative damage, is one of the most important products of lipid peroxidation and could damage the mucosa [34], while GPx is an important component of the glutathione antioxidant system, which forms an antioxidant barrier in the gut mucosa [35]. Based on the above analysis, the protective effect of LMW-ulvan on IBD damage might be attributed to an enhanced antioxidant defense system.

### 2.6. Effect of LMW-Ulvan on mRNA Expression of Colonic Epithelial Tight Junction Protein

**Figure 7:**
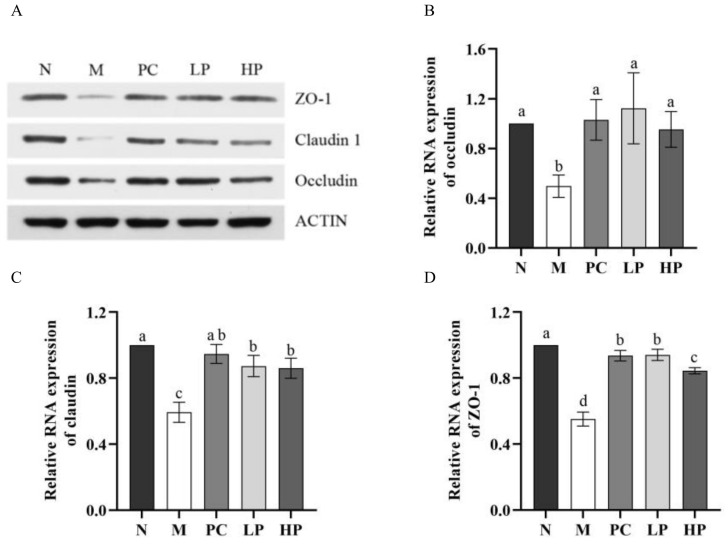
The expression of colonic epithelial tight junction protein. (**A**) Representative western blotting images for colonic epithelial tight junction protein. (**B**) The relative mRNA content of claudin. (**C**) The relative mRNA content of occludin. (**D**) The relative mRNA content of ZO-1. N: normal group, M: model, PC: positive control, LP: low dose LMW-ulvan (50 mg/kg), and HP: high dose LMW-ulvan (100 mg/kg).

To ascertain whether LMW-ulvan treatment can protect the integrity of the intestinal barrier, the expression of claudin, occluding, and tight junction protein 1 (ZO-1) was detected at both protein and mRNA level (Figure 7). The expression of three tight junction components in the M group was reduced significantly (*p* < 0.05), while the expression in the LMW-ulvan treatment groups was increased to different degrees compared with that of the N group. For ZO-1 and claudin, the expression in both HP and LP groups was significantly increased and the low dose LMW-ulvan showed a similar effect as that of 5-aminosalicylic acid (5-ASA) (Figure 7C,D). For occludin, the expression level in both HP and LP groups showed no significant difference with that of the N group (Figure 7B). Claudin, occludin, and ZO-1 are the main components of tight junction, which is the most significant structure in the intestinal mucosal mechanical barrier [10]. The tight junction can effectively prevent enteric pathogens, antigens, and other substances from entering the intestinal mucosa, preventing the activation of immune cells and abnormal immune responses and maintaining stability of mucosal barrier function and intestinal permeability [36]. The improvement of LMW-ulvan on DSS-induced colitis might be closely related to its protective effect on the intestinal mucosal barrier by increasing the expression of tight junction proteins. Thus, LMW-ulvan could further inhibit the abnormal immune response in the intestinal mucosa, thus ameliorating mucosal barrier function and intestinal mucosal permeability.

## 3. Materials and Methods

### 3.1. Materials and Reagents

*Ulva pertusa* was collected from the coast near Weihai, China, in 2019. Ulvan lyase was provided by the Applied Microbiology Laboratory (Ocean University of China). DSS (36–50 kDa) and 5-aminosalicylic acid (5-ASA) were purchased from MP Biomedicals (Solon, CA, USA) and Sigma-Aldrich (St. Louis, MO, USA), respectively. ELISA detection kits for IL-1β, IL-4, IFN-γ, malonic dialdehyde (MDA), catalase (CAT), and glutathione peroxidase (GPx) were obtained from Dakewe Biotech Corporation (Beijing, China). Primary antibodies specific for β-actin, ZO-1, occluding, and claudin-1 were purchased from Protein Tech Group (Wuhan, China). The BCA Protein Assay Kit was purchased from Beijing Solarbio Science & Technology Co., Ltd. (Beijing, China). All other reagents were of analytical grade.

### 3.2. Enzymatic Preparation and Purification of LMW-Ulvan

The ulvan was extracted according to a method described by Qiao et al. (2020) [37]. It was then degraded though an enzymatic method described by Chi et al. (2020) [20] and crude enzymatic fractions with average molecular weight of 1–5 kDa were obtained by nanofiltration membrane system. The obtained fraction was purified using ÄKTAprime plus (GE Healthcare, Woburn, MA, USA) equipped with DEAE-Sepharose CL-6B column (1.6 × 10 cm). The fraction was pre-equilibrated with ultrapure water and then eluted with a linear gradient of 0–1 M NaCl at 1.0 mL/min. The purified fraction was then dialyzed, lyophilized, and nominated as LMW-ulvan.

### 3.3. Characterization of LMW-Ulvan

The total sugar content of LMW-ulvan was determined by phenol-sulfuric acid method [38]. Sulfate content was determined after hydrolysis of trifluoroacetic acid [39]. The molecular weights of LMW-ulvan were measured according to a method described by Ye et al. (2019) [40]. The Fourier transform infrared (FI-IR) spectra of LMW-ulvan were recorded using the Magna-IR560 spectrometer (Nicolet Instrument Corp., Madison, WI, USA) according to research by Cui et al. (2019) [41]. Its monosaccharide composition was measured by reversed-phase HPLC (Agilent Technologies, Santa Clara, CA, USA) after pre-column derivatization, according to a method described by Yu et al. (2017) [22].

### 3.4. Methylation Analysis of LMW-Ulvan

Desulfation of the LMW-ulvan was achieved according to Falshaw and Furneaux (1998) [42]. Briefly, the LMW-ulvan was converted to the pyridinium salt form by dialysis against pyridinium hydrochloride (0.1 M, adjusted to pH 6.8), then against distilled water, and finally lyophilised. The resulting materials was dissolved in 89:10:1 *v*/*v* Me_2_SO-MeOH-pyridine, and heated for 4 h at 100 °C. After cooling, the desulfated product was recovered by dialysis, freeze-dried, and designated as dsLMW-ulvan.

Methylation analysis was performed as the reported method with some modification (Li et al., 2019) [23]. Briefly, 1 mg sample (LMW-ulvan or dsLMW-ulvan) dried by P_2_O_5_ was dissolved in 2.0 mL DMSO, and then 100 mg anhydrous NaH was added under nitrogen atmosphere. The mixture was stirred at 25 °C for 2 h under nitrogen atmosphere, and then 1 mL CH_3_I was added dropwise in an ice-cold water bath. The mixture was incubated in the dark at 25 °C, stirring for 3 h. Finally, 1 mL distilled water was added to terminate the reaction, and then extracted with CHCl_3_. The extract was washed with distilled water and evaporated to dryness. The methylated polysaccharide was converted into partially methylated alditol acetates, which were analysed by GC-MS (Thermo Fisher Scientific, MA, USA). The GC-MS analysis designed for methylation analysis was performed according to a report by Lin et al. (2016) [43].

### 3.5. Animals

Male C57BL/6 SPF mice (6 weeks, 18 ± 2 g) were purchased from Jinan Pengyue Experimental Animal Breeding Co., Ltd. (Shandong, China, License ID: SCXK2014-0007). The mice were adapted to a specific pathogen-free condition (12 h light/dark cycle, 22 ± 2 °C) for 7 days before the experiments, with free access to drinking water and a commercial diet. All procedures for this experiment were approved by the Animal Ethics Committee of Ocean University of China (certificate no. SYXK20120014).

### 3.6. The DSS-Induced Colitis Model

The colitis was induced by orally administering 2% (*w*/*v*) DSS drinking water for 5 days. The mice were randomly divided into five groups (*n* = 10)—the normal group (N group, drinking water), model group (M group, 2% DSS water), positive control group (PC group, 2% DSS water + 50 mg/kg 5-ASA), low dose LMW-ulvan group (LP group, 2% DSS water + 50 mg/kg LMW-ulvan), and high dose LMW-ulvan group (HP group, 2% DSS water + 100 mg/kg LMW-ulvan). The mice’s body weight, stool condition, and fecal bleeding were recorded daily. On the 13th day, the mice were sacrificed after fasting for eight hours.

### 3.7. Assessment of Severity of Colitis

The length of the colon was measured from the ileocecal junction to the anal verge [27]. The disease activity index (DAI) was calculated by average scores for changes in body weight loss, stool condition, and fecal bleeding, according to the DAI scoring system [44]. Briefly, loss in body weight was scored as follows: (i) weight loss: 0, no weight loss; 1, 1–5% loss; 2, 5–10% loss; 3, 10–15% loss; 4, more than 15% loss, (ii) stool consistency: 0, normal; 2, loose stools; 4, diarrhea, and (iii) fecal bleeding: 0, no blood; 2, positive hemoccult; 4, severe bleeding. The spleen and thymus were immediately weighted to calculate the spleen and thymus indices. Thymus or spleen index = thymus or spleen weight mg/body weight g.

### 3.8. Cytokines and Activities of Antioxidant Enzyme Assay

Blood was taken from the ocular orbit and centrifuged at 4000 rpm for 40 min at 4 °C to obtain serum. To the colon was added PBS to make 10% tissue homogenate. Then, the colonic homogenate was centrifuged at 4000 rpm for 15 min and the supernatant was taken for biochemical determination. The expression levels of the inflammatory cytokines, including IL-1β, IL-4, and IFN-γ, both in the serum and colon tissue, were determined by ELISA kits, according to the manufacture’s protocol. The level of MDA and activity of CAT and GPx in the colon were measured according to the manufacturer’s instructions.

### 3.9. Quantitative Real-Time Polymerase Chain Reaction (qRT-PCR) Analysis

Colon tissue was ground with liquid nitrogen and 10 μL β-mercaptoethanol and 500 μL Buffer GTC was added to it. Total RNA was then extracted according to the manufacturer’s instructions. The extracted RNA was reverse-transcribed into cDNA (5X All-InOneMasterMix; abm, Vancouver, Canada) and qRT-PCR amplification was performed using the SYBR Green (TOYOBO, Osaka, Japan) reagent to examine the mRNA relative expressions of ZO-1, occludin, and claudin [40]. The mRNA expression from each sample was calculated by normalizing with β-actin as an endogenous control. The primer sequences are listed in Table 2.

### 3.10. Western Blot (WB) Assay

According to the method described by Tian et al. (2020) [45], the WB procedure was as follows. Colon tissue was ground in the presence of liquid nitrogen and then lysed in RIPA lysis buffer for 30 min on ice. The lysates were centrifuged under the condition of 12,000 rpm at 4 °C for 10 min. The protein concentrations of the supernatants were detected by using a BCA protein assay kit. Then, the same amount of protein was separated by 12% SDS-PAGE and transferred to polyvinylidene difluoride (PVDF) membranes (Hybond, Sunnyvale, CA, USA) using a semidry transfer system (Bio-Rad, Hercules, CA, USA).The membranes were incubated with specific antibodies against β-actin, ZO-1,occluding, and claudin-1 overnight at 4 °C, and then HRP-conjugated secondary antibodies were incubated for 1 h at room temperature. All of the antibodies were diluted in tris buffered saline (TBS). The protein signals were analyzed using an ECL detection system (Tanon, Nanjing, China).

### 3.11. Statistics Analysis

All data were analyzed by using SPSS software 22 and expressed as the mean ± standard deviation of at least three separate experiments. Statistical significance was identified by one-way analysis of variance (ANOVA) and the Waller-Duncan test. The value of *p* < 0.05 was accepted as statistically different.

## 4. Conclusions

Our results revealed that LMW-ulvan enzymatic production of ulvan extracted from green algae U. pertusa consists of 57.23% rhamnose, 28.76% xylose, 7.42% glucuronic acid, and 1.77% glucose. Its backbone contained (1→3,4)-linked Rha, (1→4)-linked Xyl, and (1→4)-linked GlcA with small amounts of (1→4)-linked Rha residues; sulfate substitution was at C-3 of rhamnose. In addition, LMW-ulvan was able to relieve intestinal inflammation and oxidative damage caused by DSS. Meanwhile, LMW-ulvan was able to significantly increase mRNA levels of claudin, occluding, and ZO-1, which improve intestinal mucosal permeability. Consequently, the results of this study support the potential application of LMW-ulvan as a functional food ingredient for IBD.

## Figures and Tables

**Table 1:** Profile of partially *O*-methylated alditol acetates obtained by methylation analysis of LMW-ulvan.

*O*-Me-Alditol Acetate	Molar Percentage		Linkage Pattern
	LMW-Ulvan	dsLMW-Ulvan	
1,4,5-Tri-*O*-acetrl-2,3-di-*O*-metyl-L-Rha	6.65%	53.32%	→4)-Rha-(1→
1,3,4,5-Tetro-*O*-acetrl-2-*O*-metyl-L-Rha	50.12%	4.16%	→3,4)-Rha-(1→
1,4,5-Tri-*O*-acetrl-2,3-di-*O*-metyl-D-xyl	29.34%	28.45%	→4)-xyl-(1→

Desulfation of the LMW-ulvan: dsLMW-ulva, rhamnose: Rha, xylose: Xyl.

**Table 2:** Primer sequences for qRT-PCR.

Gene.	Forward	Reverse
ZO-1	CGCGGAGAGAGACAAGATGT	AAACCCAGGAGCCCTGTGAA
claudin	CAACCACAATAGCGGCATCG	GCACAGACCTGCAAGGAGAT
occludin	GATCGTGTTTGCGGATGAGC	AGCCTCCTTAGCTCGTAGTCA
β-actin	CAAGGCATTGCTGACAGGATG	TGCTGATCCACATCTGCTGG
